# Honey Lemon Alleviates Alcoholic Liver Disease via Multi-Target Synergistic Mechanisms: An Integrated Study of Network Pharmacology, Molecular Docking, and Animal Experiments

**DOI:** 10.3390/foods15081384

**Published:** 2026-04-16

**Authors:** Yaxi Zhou, Dong Xu, Meichao Bu, Ke Li, Lingyu Gao, Fei Pan, Wenjun Peng, Hualei Chen, Wenli Tian

**Affiliations:** 1 State Key Laboratory of Resource Insects, Institute of Apicultural Research, Chinese Academy of Agricultural Sciences, Beijing 100193, China; 15239407080@163.com (Y.Z.); 82101235554@caas.cn (D.X.); bmc18726625871@163.com (M.B.); 13689201711@163.com (K.L.); gaolingyu@caas.cn (L.G.); yunitcon@yeah.net (F.P.); pengwenjun@caas.cn (W.P.); 2 Beijing China-Bee Science & Technology Development Co., Ltd., Beijing 100193, China

**Keywords:** alcoholic liver disease, honey lemon water, network pharmacology, molecular docking, inflammatory response, oxidative stress

## Abstract

Honey lemon (H&L) is a traditional beverage known for its potential liver-protective effects, but its mechanisms against alcoholic liver disease (ALD) remain poorly understood. This study aimed to investigate the hepatoprotective properties of H&L and explore its multi-target mechanisms in alleviating ALD. Using network pharmacology and molecular docking, we identified 26 bioactive compounds in H&L and 335 potential targets associated with ALD. Pathway enrichment analysis revealed that H&L might exert its influence by regulating inflammation, oxidative stress and ethanol metabolism. Molecular docking further demonstrated strong binding interactions between key flavonoids (hesperidin, diosmin, and eriocitrin) and crucial targets, such as AKT1, SRC, STAT3, as well as ethanol-metabolizing enzymes like ADH, ALDH, and CYP2E1. In vivo experiments suggested that H&L alleviated liver injury and significantly improved selected indicators related to ethanol metabolism, oxidative stress, and inflammatory response. For several variables, including ALT/AST, ALDH, IL-6, and hepatic ethanol content, improvement trends were observed, although not all differences reached statistical significance. Overall, the results suggest that the protective effect of H&L against ALD may be associated with a multi-component, multi-target, and multi-pathway mode of action, supporting its potential for further investigation as a functional food candidate.

## 1. Introduction

Alcoholic liver disease (ALD) is a chronic liver injury syndrome caused by long-term or excessive alcohol consumption and is one of the leading causes of liver cirrhosis and hepatocellular carcinoma worldwide [1]. Clinically, ALD presents a spectrum of manifestations, ranging from simple steatosis and alcoholic hepatitis to fibrosis and ultimately cirrhosis [2]. Epidemiological studies have shown that the incidence of ALD is steadily increasing due to the growing prevalence and younger age trend of alcohol consumption, placing a substantial burden on public health systems [3,4].

The pathogenesis of ALD is highly complex, with oxidative stress, immune-inflammatory responses, lipid metabolism disorders, and the accumulation of toxic ethanol metabolites identified as major contributing factors [5,6]. Extensive research indicates that ethanol metabolism produces acetaldehyde and reactive oxygen species (ROS), which can directly damage hepatocyte membranes, induce lipid peroxidation and mitochondrial dysfunction, activate Kupffer cells and the NF-κB signalling pathway, and subsequently trigger the release of inflammatory cytokines such as tumour necrosis factor-alpha (TNF-α), interleukin-6 (IL-6), and interleukin-1 beta (IL-1β), leading to sustained liver tissue damage [7,8,9].

Natural products, especially bioactive compounds derived from dietary sources, have attracted increasing attention for their multi-targeted activities and favourable safety profiles in the prevention and adjunctive treatment of chronic diseases [10]. Honey and lemon are two widely consumed natural foods with long histories of traditional use in various cultural contexts for health maintenance and physical conditioning. Honey is rich in polyphenols, flavonoids, vitamins, and organic acids, demonstrating strong antioxidant, anti-inflammatory, and immunomodulatory properties [11,12]. Lemon contains abundant citric acid, eriocitrin, hesperidin, and vitamin C, and exhibits beneficial effects in regulating gastrointestinal function, improving redox status, and exerting antimicrobial and anti-inflammatory actions [13,14]. Notably, previous studies have shown that lemon juice can protect against alcohol-induced liver damage in mice [15].

In recent years, honey lemon (H&L), a natural beverage made from honey and lemon, has gained popularity as a functional drink due to its refreshing taste, simple preparation, and natural origin. Anecdotal reports suggest that H&L may help relieve alcohol-related discomfort and promote recovery. However, although honey and lemon individually have been reported to show antioxidant, anti-inflammatory, and hepatoprotective activities, whether their combined formulation exerts protective effects against alcoholic liver disease remains insufficiently studied. In particular, the potential active components, putative molecular targets, and the possible relationship among ethanol metabolism, oxidative stress, and inflammatory regulation in H&L intervention remain unclear. These gaps require systematic investigation.

Traditional pharmacological approaches often face limitations when investigating complex natural beverages like H&L, which involve multiple components, targets, and pathways. These limitations include long research cycles, high costs, and a tendency to focus on single mechanisms. In recent years, the development of network pharmacology and molecular docking has provided innovative strategies for studying the mechanisms of natural products [16,17]. Network pharmacology integrates omics data, bioinformatics, and systems biology to systematically reveal the interaction networks between complex compounds and disease-related targets. Molecular docking, on the other hand, simulates the binding patterns of bioactive compounds and target proteins at the molecular level, allowing for quantitative assessment of binding affinities and interaction sites, thus offering precise structural insights into mechanism prediction. The combination of these two methods has been widely applied to investigate the pharmacological mechanisms of traditional Chinese medicine, compound extracts, and functional beverages [18,19].

Against this background, this study focused on H&L as the research subject, aiming to evaluate its protective effect against ALD and explore its potential mechanisms. By integrating network pharmacology and molecular docking strategies, and validating findings through animal experiments, we explored the potential protective effects of H&L against ALD and investigated its possible mechanisms using an integrated strategy. The results not only enhance our understanding of H&L’s mechanism of action but also provide a theoretical foundation for developing natural multi-target interventions based on common dietary resources, thereby contributing to non-pharmacological nutritional strategies for chronic liver disease management.

## 2. Materials and Methods

### 2.1. Materials

The commercially available H&L used in this study was provided by Beijing Zhongmi Technology Development Co., Ltd. (Beijing, China). The product consisted of water and a honey–lemon concentrate supplied separately, which were mixed and shaken immediately before use according to the product form. The supplier was not involved in the experimental design, data analysis, interpretation of results, manuscript writing, or publication decision. The sugar composition and basic parameters of H&L are provided in Supplementary Information (Tables S1 and S2). The 56 °C Red Star Erguotou liquor was purchased from a local supermarket. Assay kits for aspartate aminotransferase (AST), alanine aminotransferase (ALT), alcohol dehydrogenase (ADH), aldehyde dehydrogenase (ALDH), catalase (CAT), superoxide dismutase (SOD), and malondialdehyde (MDA) were obtained from Nanjing Jiancheng Bioengineering Institute (Nanjing, China). Ethanol content and cytochrome P450 2E1 (CYP2E1) assay kits were purchased from Shanghai Jianglai Biotechnology Co., Ltd. (Shanghai, China). Enzyme-linked immunosorbent assay (ELISA) kits for TNF-α, IL-6, and IL-1β were purchased from Hangzhou Lianke Biotechnology Co., Ltd. (Hangzhou, China). Unless otherwise stated, all other reagents used were of analytical grade.

### 2.2. Network Pharmacology

#### 2.2.1. Identification of Major Components and Potential Targets of H&L

The main active ingredients of H&L were collected from published literature and online databases. After manual screening, 26 compounds were included, and the results are shown in Table S3. The SMILES identifiers of these compounds were retrieved from the PubChem database (https://pubchem.ncbi.nlm.nih.gov/) (accessed on 27 September 2025). Target prediction was then conducted using the SwissTargetPrediction platform (http://www.swisstargetprediction.ch/) (accessed on 27 September 2025), specifying Homo sapiens as the species. Targets with a probability value of 0 were excluded, and the remaining targets were merged for further analysis (Table S4).

#### 2.2.2. Acquisition of Therapeutic Targets for ALD

Potential targets related to ALD were obtained from the GeneCards database (https://www.genecards.org/) (accessed on 27 September 2025) [20]. The keywords used for searching were “Alcoholic liver disease (ALD)”, “Acute alcoholic liver disease (AALD)”, and “Chronic alcoholic liver disease (CALD)”. Targets with a relevance score of less than 10 were excluded. The target lists from ALD, AALD, and CALD were then merged, duplicates removed, and a total of 5051 targets were retained as potential ALD-related therapeutic targets.

#### 2.2.3. Construction of Compound-Target Interaction Network

Duplicate targets of H&L were removed. The information of H&L components and their corresponding targets was then imported into Cytoscape 3.10.3 [21] to construct a component–target interaction network. Edges in the network represent interactions between compounds and targets.

#### 2.2.4. Screening of Potential Targets of H&L in ALD Treatment

Venny 2.1.0 (https://bioinfogp.cnb.csic.es/tools/venny/) (accessed on 27 September 2025) was used to analyze the intersection between predicted targets of H&L and the ALD-related targets. A total of 335 overlapping targets were identified as potential intervention targets (Table S5).

#### 2.2.5. GO and KEGG Enrichment Analysis

Gene Ontology (GO) and Kyoto Encyclopedia of Genes and Genomes (KEGG) enrichment analyses were performed using the DAVID online platform (https://davidbioinformatics.nih.gov/) (accessed on 27 September 2025), with *p* < 0.05 considered statistically significant. The 335 intersecting genes were uploaded using the “Functional Annotation” tool (Identifier: Official Gene Symbol; List Type: Gene List). The top 20 entries of Molecular Function (MF), Cellular Component (CC), Biological Process (BP), and KEGG pathways were exported for further analysis. Visualizations of MF, CC, BP, and KEGG results were generated using the online Bioinformatics platform (http://www.bioinformatics.com.cn/) (accessed on 27 September 2025) [22].

#### 2.2.6. Protein–Protein Interaction (PPI) Network Construction and Hub Gene Screening

The 335 overlapping genes were uploaded to the STRING database (https://cn.string-db.org/) (accessed on 27 September 2025) [23] using the “Multiple Proteins” function and “Homo sapiens” as the species. TSV-format data were downloaded and imported into Cytoscape 3.10.3. The CytoHubba plugin was used to analyze the PPI network using five algorithms: Degree, MCC (Maximal Clique Centrality), MNC (Maximum Neighbourhood Component), EPC (Edge Percolated Component), and Closeness. The top 15 hub genes from each algorithm were obtained, and their intersections were visualized using Draw Venn Diagram (https://bioinformatics.psb.ugent.be/webtools/Venn/) (accessed on 27 September 2025), resulting in eight core hub genes. The PPI network of these eight hub genes was reconstructed via STRING (Tables S6 and S7).

### 2.3. Molecular Docking and Heatmap Analysis

The main active components of H&L were used as ligands. Ligand structures were downloaded in SDF format from the PubChem database (https://pubchem.ncbi.nlm.nih.gov/) (accessed on 30 September 2025) and converted to PDB format using PyMOL (Version 3.10). Geometry optimization was performed using Avogadro software (Version 2.0.0) under the MMFF94s force field to eliminate unfavourable bond lengths and angles [24]. The eight hub proteins (EGFR, SRC, CTNNB1, STAT3, AKT1, PIK3CA, STAT1, ERBB2) and three ALD-related enzymes (ADH, ALDH, and CYP2E1) were used as receptors (Table S8). Protein structures were retrieved from UniProt (https://www.uniprot.org/) (accessed on 30 September 2025) and downloaded from the PDB database (https://www.rcsb.org/) (accessed on 30 September 2025) in PDB format. PyMOL was used to remove water molecules and heteroatoms.

AutoDockTools 1.5.7 (Version 1.5.7) was used to process both ligands and receptors by adding hydrogens, calculating Gasteiger charges, and merging nonpolar hydrogens [25]. Files were saved in PDBQT format [26]. The GetBox plugin for PyMOL (https://github.com/MengwuXiao/Getbox-PyMOL-Plugin) (accessed on 30 September 2025) was used to define docking grid boxes. Semi-flexible molecular docking was performed using AutoDock Vina [27], and docking results were visualized and analyzed with PyMOL [28]. Binding affinity was evaluated by docking energy, with values less than −5 kcal/mol considered to indicate moderate or strong binding.

The docking poses and energies were extracted using custom scripts, and the best binding energies were used to generate a ligand-target heatmap. Heatmap visualization was performed using Origin 2024.

### 2.4. Animals and Experimental Design

#### 2.4.1. Animal Model and Experimental Grouping

A total of 24 healthy male Kunming mice (25 ± 2 g) were purchased from Beijing Huafukang Bioscience Co., Ltd. (Beijing, China). The animals were housed in a specific pathogen-free (SPF) facility at Beijing Langke Biotechnology Co., Ltd. (Beijing, China). All animal experiments were approved by the Institutional Animal Care and Use Committee (IACUC), with the ethical approval number: IACUC-20250608-01. The mice were maintained under standard laboratory conditions at a temperature of 22–25 °C and relative humidity of 50–55%, with free access to food and water. After one week of acclimatization, the mice were randomly divided into three groups (*n* = 8 per group): control group (CON), model group (MOD), and honey lemon group (H&L). The treatments were as follows: the CON group received an equivalent volume of physiological saline via oral gavage; The MOD group was pretreated with an equivalent volume of saline and served as the model control group. This group was included to provide a baseline under the same ethanol-challenge conditions and to exclude the possible influence of the gavage procedure itself; and the H&L group received freshly prepared H&L (2 g/kg body weight). Pretreatment was performed once daily for three consecutive days. During pretreatment, all mice had free access to standard feed and water.

After pretreatment, acute alcoholic liver injury was induced in all groups except the CON group following a previously reported protocol [29]. Specifically, 56% (*v*/*v*) Red Star Erguotou liquor was administered via oral gavage at a dose of 12 mL/kg body weight, once every 12 h for three consecutive times. The CON group received an equal volume of saline. Four hours after the final ethanol administration, mice were euthanized by cervical dislocation. Blood was collected from the ocular cavity, centrifuged at 3000 rpm for 10 min to obtain serum, and stored at −20 °C for analysis. The liver and spleen were dissected, blotted to remove residual blood, and weighed to calculate organ indices. Liver tissue was sectioned; one portion was fixed in 4% neutral formalin for histological analysis, and the remaining portion was snap-frozen at −80 °C for biochemical assays. Owing to sample damage or insufficient material during collection and processing, the final analyses were conducted using 6 mice per group.

#### 2.4.2. Organ Index Calculation

Body weight was recorded throughout the experiment. The fresh liver and spleen weights of each mouse were measured upon sacrifice. The liver and spleen indices were calculated as follows [30,31]: Liver index = (Liver weight/Body weight) × 100%; Spleen index = (Spleen weight/Body weight) × 100%.

#### 2.4.3. Histological Examination (H&E Staining)

Liver tissue fixed in 4% paraformaldehyde was embedded in paraffin and sectioned at 4 μm. Sections were deparaffinized and stained with haematoxylin for 5 min, followed by differentiation in 1% hydrochloric acid ethanol, bluing under running water, and eosin staining for 2 min. After gradient ethanol dehydration and xylene clearing, sections were mounted using neutral resin and examined under a light microscope (JEM1400, Tokyo, Japan) [32]. Furthermore, we conducted histopathological scoring on the liver sections, and the scoring table is shown in Table S9.

#### 2.4.4. Measurement of Liver Function-Related Enzymes

The activities of ALT and AST in liver tissue were determined using commercial kits (Nanjing Jiancheng Bioengineering Institute) (Beijing, China) based on enzymatic kinetics and expressed as U/mg protein. The activities of ADH, ALDH, and CYP2E1 in liver tissue, as well as the serum levels of TNF-α, IL-6, and IL-1β, were measured using conventional and ELISA kits according to the manufacturers’ protocols.

#### 2.4.5. Determination of Ethanol Content in Serum and Liver

Ethanol concentrations in serum and liver were determined using ethanol detection kits from Shanghai Jianglai Biotechnology Co., Ltd. (Shanghai, China).

#### 2.4.6. Assessment of Oxidative Stress Markers

Commercial kits were used to assess SOD activity, CAT activity, and MDA content in liver homogenates. SOD and CAT are essential antioxidant enzymes, while MDA is a marker of lipid peroxidation. Together, they reflect the oxidative stress status of the organism [6]. All measurements were performed according to the kit instructions and analyzed using a microplate reader. Enzymatic activities and metabolite levels were calculated based on standard curves.

### 2.5. Statistical Analysis

All experimental data are expressed as mean ± standard deviation (SD). One-way analysis of variance (ANOVA) was performed using PSPP software (Version 2.0.0-g5b54d1), followed by Duncan’s multiple range test for pairwise comparisons. Differences were considered statistically significant at *p* < 0.05. Distinct lowercase letters were used to indicate significant differences between groups.

## 3. Results and Discussion

### 3.1. Intersection of Component-Target, and Identification of Core Targets

To investigate the potential pharmacological mechanisms by which H&L exerts protective effects against ALD, we employed a network pharmacology strategy to systematically predict and analyze its active compounds and targets. A total of 26 representative bioactive compounds in H&L—including flavonoids, polyphenols, organic acids, and amino acids—were identified from the literature and online databases (Table S3). Target prediction via the SwissTargetPrediction platform yielded 450 non-redundant potential targets.

**Figure 1:**
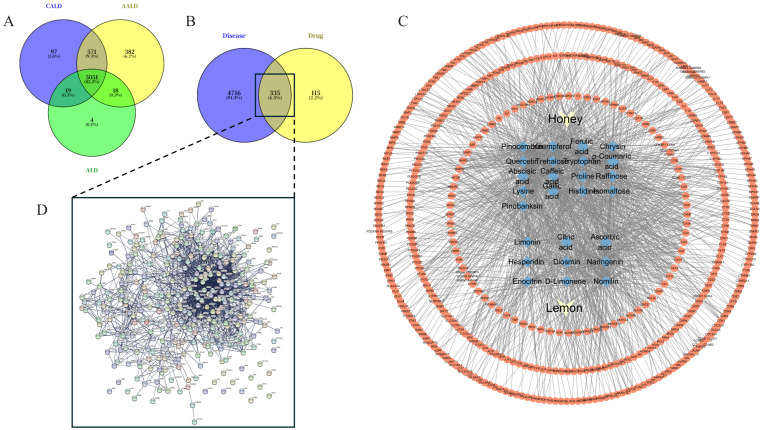
Intersection analysis of H&L targets and ALD-related targets, and construction of PPI and component–target networks. (**A**) Acquisition of disease core targets; (**B**) Acquisition of intersection targets between diseases and drug targets; (**C**) Network diagram of the interaction between components and targets; (**D**) PPI network of 335 intersection targets.

For disease-related targets, after removing duplicates, a total of 5051 ALD-related targets were identified (Figure 1A), providing the disease background for subsequent intersection analysis. The intersection of the 450 H&L-related targets and the 5051 ALD-related targets yielded 335 overlapping genes (Figure 1B), which were considered key candidates for H&L intervention in ALD.

These 335 targets were input into the STRING database to construct a protein–protein interaction (PPI) network (Figure 1D). The resulting network exhibited high density and strong connectivity, suggesting functional coordination among these proteins that may underline the therapeutic potential of H&L.

To further elucidate the interaction patterns between components and targets, a component-target network was visualized using Cytoscape (Figure 1C). Key components such as hesperidin, diosmin, eriocitrin, pinocembrin, and kaempferol were found to interact with multiple targets, reflecting the multitarget regulatory potential of H&L. The network topology indicated both overlapping and complementary target spectra between honey- and lemon-derived compounds, suggesting that honey- and lemon-derived compounds may jointly contribute to the observed protective effects of H&L.

### 3.2. GO and KEGG Pathway Enrichment Analysis

**Figure 2:**
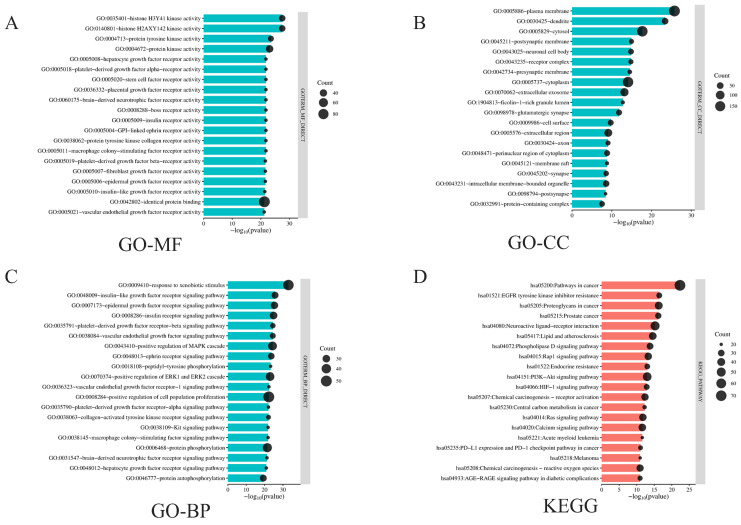
GO and KEGG enrichment analysis of the overlapping genes. (**A**) Enriched molecular function (MF) terms; (**B**) Enriched cellular component (CC) terms; (**C**) Enriched biological process (BP) terms; (**D**) KEGG pathway enrichment analysis results. The X-axis represents statistical significance (−log_10_(*p*-value)), the Y-axis shows the enriched terms, and the size of the dots indicates the number of involved targets (Count).

The 335 overlapping targets were subjected to GO and KEGG enrichment analysis using the DAVID platform. GO terms were categorized into MF, CC, and BP [33]. A total of 919BPs, 107CCs, and 288MFs were enriched (Table S10). The top 20 terms in each category are shown in Figure 2A–C.

In the MF category (Figure 2A), significant enrichment was observed in protein kinase activity (especially tyrosine kinases and histone-modifying kinases) and growth factor receptor binding, suggesting strong involvement in signal transduction and cell regulation. CC enrichment (Figure 2B) revealed predominant localization of targets to the plasma membrane and membrane-associated regions, indicating a role in transmembrane signalling. Enrichment in neuronal structures, synapses, and extracellular vesicles further suggests involvement in neuro-signalling, secretion, and immune interactions. BP enrichment (Figure 2C) showed that genes were mainly involved in signalling pathways related to growth factor receptors (e.g., IGF, EGF, PDGF, VEGF), protein tyrosine phosphorylation, MAPK/ERK cascades, xenobiotic response, and immune regulation. These findings indicate that H&L may influence multiple physiological and pathological processes through modulation of cellular signalling, stress response, and proliferation [34].

In addition, KEGG has enriched 178 signalling pathways (Table S11). According to the KEGG pathway enrichment results shown in Figure 2D, the overlapping genes were significantly enriched in multiple pathways, such as “Pathways in cancer,” “EGFR tyrosine kinase inhibitor resistance,” “Proteoglycans in cancer,” and “Melanoma,” suggesting that these genes may play critical roles in cancer-related signal regulation, immune evasion, or drug resistance mechanisms. Additionally, several classical signalling pathways, including PI3K-Akt, Ras, Rap1, calcium signalling, and HIF-1 signalling pathways, were also significantly enriched, indicating their broad involvement in the regulation of cell proliferation, differentiation, and survival [35]. These findings implied that the identified genes may have potential functions in inflammation and metabolic disorders. Overall, the KEGG enrichment results provide important insights into the molecular mechanisms and potential therapeutic targets of related diseases.

### 3.3. PPI Network Analysis and Bub Gene Identification

**Figure 3:**
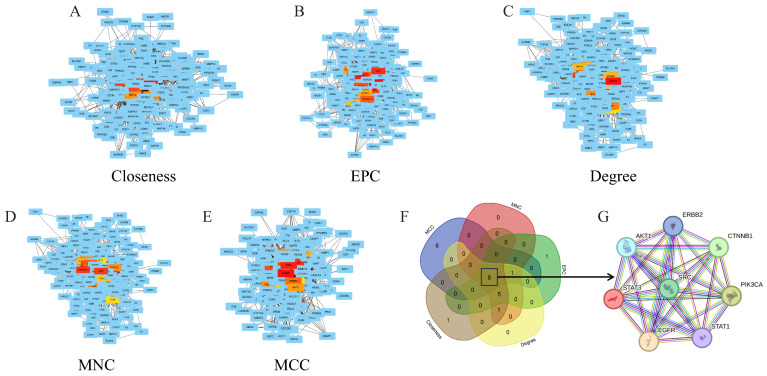
PPI network of overlapping genes analyzed using five CytoHubba algorithms (**A**–**E**), Venn intersection of top-ranked genes (**F**), and PPI network of eight hub genes (**G**).

To identify hub genes involved in ALD regulation, the STRING database was used to construct a comprehensive PPI network, followed by analysis with five ranking algorithms (Degree, MCC, Closeness, EPC, and MNC) using the CytoHubba plugin in Cytoscape [36]. The top 15 hub genes from each algorithm were visualized in Figure 3A–E, with red and yellow nodes representing genes with the highest centrality scores. High consistency was observed among algorithms, with genes such as EGFR, AKT1, and MAPK1 frequently ranking at the core of the network. These genes are considered crucial regulators of the network and likely contributors to the biological effects of H&L. A Venn diagram intersection across all five algorithms revealed eight common hub genes: EGFR, AKT1, PIK3CA, SRC, STAT1, STAT3, CTNNB1, and ERBB2 (Figure 3F, Table S8). Their interactions were further visualized via STRING (Figure 3G), indicating strong interconnectivity and functional cooperation in ALD modulation. A similar study identified 15 candidate genes involved in garlic-mediated ALD intervention [37], and notably, AKT1 was also a shared target in our analysis, reinforcing its potential as a key therapeutic target.

### 3.4. Molecular Docking Heatmap Reveals Multitarget Potential of H&L

**Figure 4:**
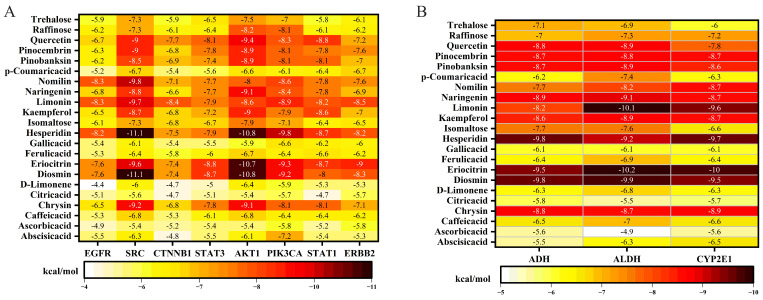
Heatmaps of binding affinities between active compounds and (**A**) eight hub proteins and (**B**) three key ethanol-metabolizing enzymes.

To further evaluate the molecular basis of H&L in ALD intervention, 22 representative compounds were docked with eight hub proteins (EGFR, SRC, CTNNB1, STAT3, AKT1, PIK3CA, STAT1, ERBB2) and three alcohol metabolism-related enzymes (ADH, ALDH, CYP2E1). Binding affinities (in kcal/mol) are presented as heatmaps in Figure 4 and detailed in Table S12.

As shown in Figure 4A, most compounds exhibited favourable binding affinities (<−5 kcal/mol) [38]. Flavonoids such as hesperidin, diosmin, and eriocitrin showed strong binding across multiple targets, with several interactions exceeding −9 kcal/mol. Notably, SRC and AKT1 had the lowest binding energies with multiple ligands, suggesting key roles in H&L-mediated regulation of ALD. For instance, hesperidin and SRC showed a binding energy of −11.1 kcal/mol, while diosmin and AKT1 reached −10.8 kcal/mol, indicating high-affinity interactions. For ethanol-metabolizing enzymes (Figure 4B), diosmin, eriocitrin, limonin, and hesperidin showed strong binding to ADH, ALDH, and CYP2E1. For example, eriocitrin bound ALDH with an affinity of −10.2 kcal/mol, suggesting these compounds may enhance alcohol metabolism and reduce toxic metabolite accumulation, thereby contributing to hepatoprotection [39]. In summary, the docking results support a multitarget interaction model for H&L in ALD, involving key pathways such as EGFR, PI3K-Akt, and STAT signalling, as well as modulation of ethanol metabolism. These findings provide structural and mechanistic support for the use of H&L as a natural functional beverage in the prevention and management of ALD.

### 3.5. Visualization and Analysis of Molecular Docking Results

**Figure 5:**
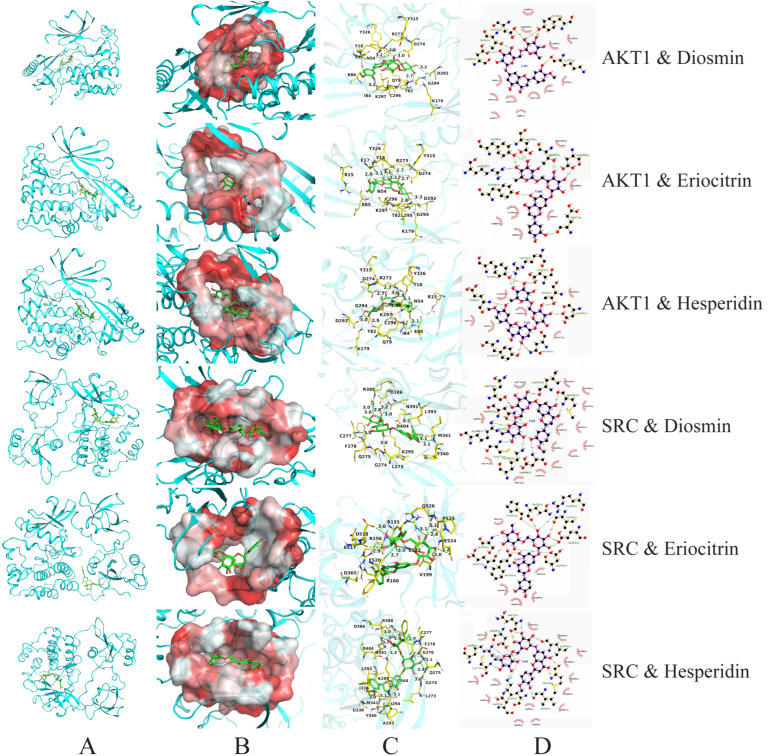
Molecular docking visualization of AKT1 and SRC with diosmin, eriocitrin, and hesperidin. (**A**) Protein–ligand complex; (**B**) Ligand in hydrophobic pocket; (**C**) 3D interaction diagram; (**D**) 2D interaction diagram.

**Figure 6:**
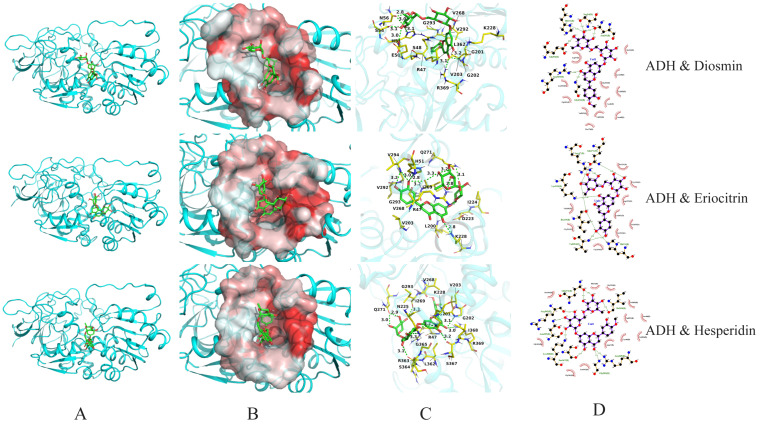
Molecular docking visualization of ADH with diosmin, eriocitrin, and hesperidin. (**A**) Protein–ligand complex; (**B**) Ligand in hydrophobic pocket; (**C**) 3D interaction diagram; (**D**) 2D interaction diagram.

**Figure 7:**
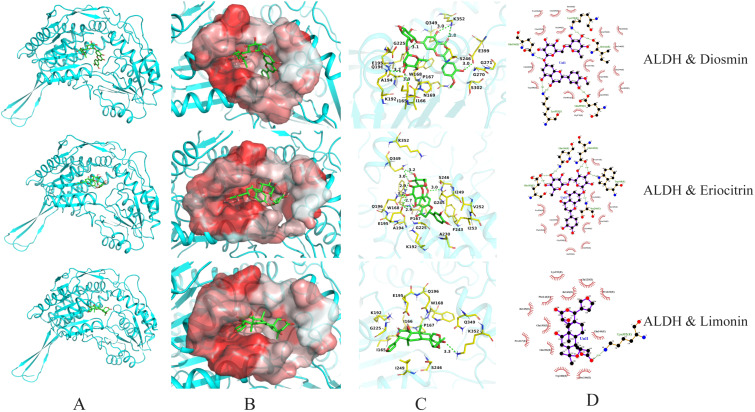
Molecular docking visualization of ALDH with diosmin, eriocitrin, and limonin. (**A**) Protein–ligand complex; (**B**) Ligand in hydrophobic pocket; (**C**) 3D interaction diagram; (**D**) 2D interaction diagram.

**Figure 8:**
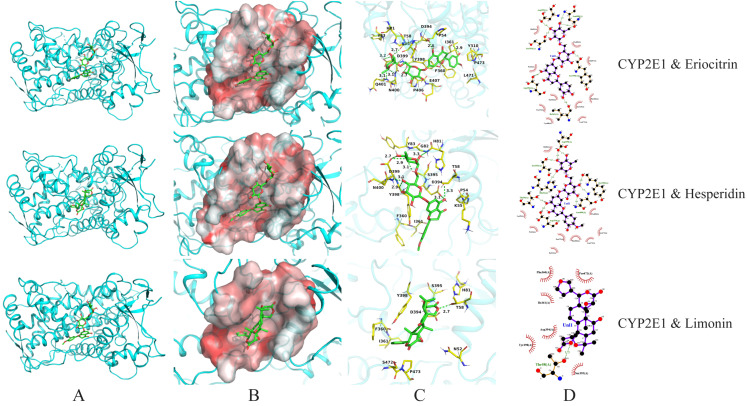
Molecular docking visualization of CYP2E1 with eriocitrin, hesperidin, and limonin. (**A**) Protein–ligand complex; (**B**) Ligand in hydrophobic pocket; (**C**) 3D interaction diagram; (**D**) 2D interaction diagram.

To further investigate the interaction mechanisms between key active components (particularly diosmin, eriocitrin, hesperidin, and limonin) and multiple target proteins, molecular docking visualizations were performed based on binding energy data. The binding behaviours with five key proteins (AKT1, SRC, ADH, ALDH, and CYP2E1) were analysed. The results are shown in Table 1 and Figure 5, Figure 6, Figure 7 and Figure 8.

In the AKT1 docking results, diosmin, eriocitrin, and hesperidin all exhibited strong binding affinities ranging from −10.7 to −10.8 kcal/mol, indicating thermodynamically stable interactions (Figure 5). Diosmin formed six hydrogen bonds, with key residues including Asp274, Thr82, Tyr18, Asp292, and Lys297, most of which are located within AKT1’s kinase domain, playing critical roles in phosphorylation regulation [40]. Eriocitrin and hesperidin formed 8 and 9 hydrogen bonds, respectively, frequently interacting with Asp274, Tyr18, Thr82, Arg273, Asp292, and Tyr326, suggesting these residues are vital for ligand recognition. These results imply that flavonoids in H&L may interfere with AKT1 signalling by stably binding within its active pocket.

In the case of the SRC target, both diosmin and hesperidin exhibited binding energies of −11.1 kcal/mol, representing the strongest affinities among all tested ligands in this study (Figure 5). Diosmin formed 12 hydrogen bonds, while hesperidin formed as many as 14 hydrogen bonds, primarily interacting with residues such as Leu273, Met341, Asp404, Cys277, Arg388, Asn391, and Gln275. These amino acids are widely distributed across the SH2 and kinase domains of SRC, suggesting that they may collectively maintain the stability of the ligand–protein complex through an extensive hydrogen bond network. Although eriocitrin showed a slightly weaker binding energy (−9.6 kcal/mol), it still formed 11 hydrogen bonds with residues such as Glu524, Asp518, Pro525, and Ser522, which may be located in the regulatory region of SRC [16]. Overall, the multiple hydrogen bond interactions and low binding energies between SRC and the flavonoid ligands indicate that SRC is likely a key binding target.

For the ADH target, all three ligands demonstrated binding energies in the range of −9.5 to −9.8 kcal/mol. Hesperidin formed the highest number of hydrogen bonds (9), interacting with residues such as His51, Asn56, Glu50, Arg369, and Gly202, which are predominantly located within the catalytic centre of ADH. This suggests that hesperidin may exert its effects via a competitive inhibition mechanism (Figure 6). Both eriocitrin and diosmin formed 7 hydrogen bonds each, with interacting residues such as Ser54, Ile269, Gln271, and Lys228 also widely distributed within the structural core of ADH [41].

Docking with ALDH revealed small variations in binding energy (−9.9 to −10.2 kcal/mol). Eriocitrin showed the strongest binding (−10.2 kcal/mol), forming 7 hydrogen bonds with residues such as Gln196, Glu195, and Ser246, likely located near the enzyme’s cofactor-binding site [42] (Figure 7). Diosmin and limonin formed fewer hydrogen bonds (6 and 1, respectively), suggesting weaker or shallower binding.

For CYP2E1, eriocitrin and hesperidin exhibited strong affinities (−10.0 and −9.7 kcal/mol) and formed 7 and 9 hydrogen bonds, respectively, interacting with residues including Asp399, Asn400, Ser395, and Tyr398—located within the substrate channel and catalytic centre of CYP2E1 [43,44] (Figure 8). In contrast, limonin formed only one hydrogen bond with Thr58 and had the weakest binding affinity (−9.6 kcal/mol), indicating a possibly weak or nonspecific interaction.

Overall, hesperidin demonstrated the strongest and most extensive binding across multiple targets, characterized by low binding energies and extensive hydrogen bond networks, particularly with AKT1 and SRC, suggesting its potential role in modulating oncogenic signalling pathways. Key factors contributing to its high binding efficacy include favourable binding energy, broad residue distribution, and multiple hydrogen bonds. Diosmin and eriocitrin also showed promising multi-target binding properties, underscoring the structural advantages of flavonoids in protein–ligand recognition. Collectively, these findings highlight key molecular interactions and offer valuable guidance for pharmacological exploration and lead optimization.

### 3.6. H&L Alleviates Alcohol-Induced Liver Injury

**Figure 9:**
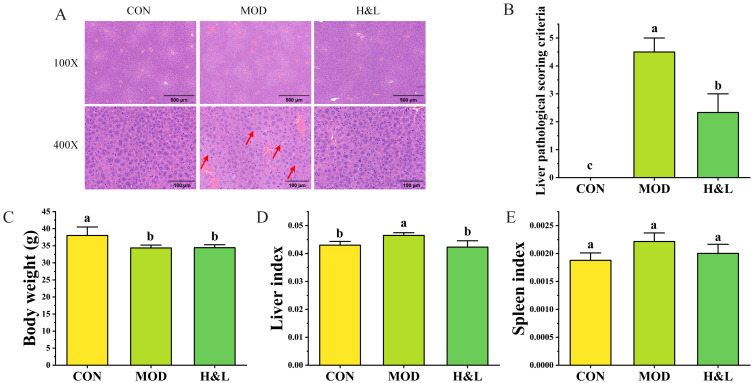
Effects of H&L on ethanol-induced liver injury (*n* = 6). (**A**) Representative H&E-stained liver sections of mice from each group (magnification: 100×, 400×). (**B**) Liver pathological score. (**C**) Body weight of mice in each group. (**D**) Liver index. (**E**) Spleen index. Distinct lowercase letters were used to indicate significant differences between groups.

To evaluate the protective effect of H&L against ALD, an acute alcohol-induced liver injury model was established in mice. H&E staining was performed to observe histopathological changes in liver tissues across groups (Figure 9A). Figure 9B shows the results of liver pathological scoring. In the CON group, hepatocytes exhibited intact morphology and orderly arrangement without any notable pathological changes. In contrast, the MOD group showed pronounced vacuolar degeneration of hepatocytes, dilated hepatic sinusoids, and significant infiltration of inflammatory cells, indicating that ethanol administration successfully induced acute liver injury (as indicated by red arrows). Notably, the H&L group displayed near-normal hepatocyte morphology, significantly reduced inflammatory cell infiltration, and alleviated steatosis, suggesting a strong hepatoprotective effect of H&L [15].

In terms of body weight (Figure 9B), mice in the MOD group exhibited a significant reduction compared to the CON group (*p* < 0.05). H&L treatments did not significantly reverse this trend, possibly due to reduced food intake or the short experimental duration. As shown in Figure 9C, liver index values were significantly elevated in the MOD group, indicating hepatic swelling. H&L treatments reduced the liver index (*p* < 0.05), suggesting an effective alleviation of liver enlargement. Regarding spleen index (Figure 9D), the MOD group exhibited a marked increase, reflecting heightened immune stress. H&L treatment significantly reduced the spleen index, restoring it to a level comparable with the control group.

Collectively, these findings indicate that H&L effectively ameliorates ethanol-induced hepatic histological damage, abnormal liver and spleen organ indices, and other pathological manifestations, thereby demonstrating a promising protective effect against acute alcoholic liver injury [45].

### 3.7. Effects of H&L on Transaminase Levels and Ethanol Metabolism

**Figure 10:**
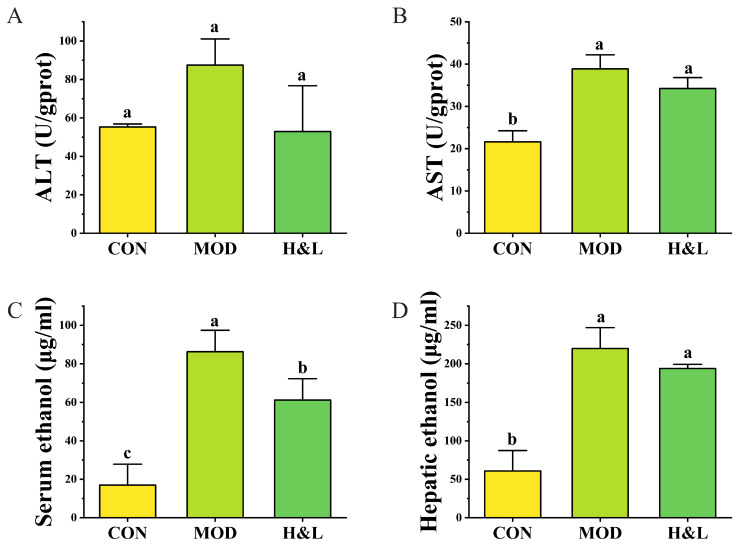
Effects of H&L on transaminase levels and ethanol accumulation in mice (*n* = 6). (**A**) ALT levels in liver tissue; (**B**) AST levels in liver tissue; (**C**) Ethanol concentration in serum; (**D**) Ethanol concentration in liver tissue. Distinct lowercase letters were used to indicate significant differences between groups.

To further evaluate the hepatoprotective effect of H&L against ethanol-induced liver injury, ALT and AST levels in liver tissue, as well as ethanol concentrations in serum and liver tissues, were measured. Regarding liver function, the MOD group exhibited significantly elevated levels of ALT and AST (*p* < 0.05; Figure 10A,B), indicating substantial hepatocellular damage and enzyme leakage due to ethanol exposure. H&L groups showed downward trends in ALT and AST levels. Although the reduction was not statistically significant, it suggests a potential protective effect of H&L in alleviating liver cell injury.

In terms of ethanol accumulation, the MOD group exhibited significantly higher serum ethanol concentrations (Figure 10C). Treatment with H&L significantly reduced serum ethanol levels (*p* < 0.05), with a more pronounced reduction observed in the H&L group. This suggests that H&L may exert a “sobering” effect by promoting ethanol metabolism or slowing its absorption. Hepatic ethanol levels (Figure 10D) were also significantly increased in the MOD group, indicating ethanol accumulation in the liver. Although H&L treatment significantly decreased hepatic ethanol content, a downward trend was observed, indicating its potential to reduce intrahepatic ethanol burden [46].

In summary, H&L may alleviate alcohol-induced liver injury by reducing ALT levels and lowering ethanol residues, particularly in serum. Its ability to facilitate ethanol clearance may be related to modulation of the ADH/ALDH metabolic system or enhancement of hepatic antioxidant capacity.

### 3.8. Regulatory Effects of H&L on the Activities of Ethanol-Metabolizing Enzymes ADH, ALDH, and CYP2E1

**Figure 11:**
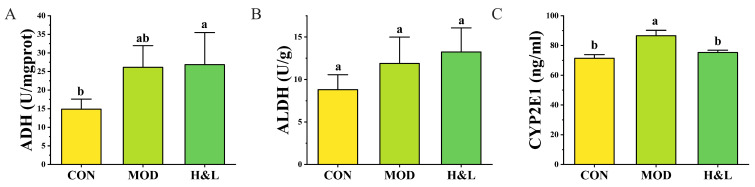
Activities of alcohol-metabolizing enzymes in liver tissue (*n* = 6). (**A**) Alcohol dehydrogenase (ADH); (**B**) Aldehyde dehydrogenase (ALDH); (**C**) Cytochrome P450 2E1 (CYP2E1). Distinct lowercase letters were used to indicate significant differences between groups.

The activity levels of ADH, ALDH, and CYP2E1 in liver tissue are directly involved in ethanol metabolism. We further quantified the enzymatic activities of these proteins using ELISAs, and the results are presented in Figure 11.

As shown in Figure 11A, ADH activity in the MOD group was moderately increased compared to the CON group, though the difference was not statistically significant, suggesting that ethanol exposure may upregulate ADH expression. H&L groups exhibited higher ADH activity than the CON group (*p* < 0.05), indicating that H&L may promote the primary phase of ethanol metabolism by enhancing the conversion of ethanol to acetaldehyde. Regarding ALDH activity (Figure 11B), all treatment groups (MOD, and H&L) showed increased activity compared to the CON group, although the differences were not statistically significant. This suggests that under ethanol stress, there may be a compensatory upregulation of ALDH to facilitate acetaldehyde clearance. For CYP2E1 expression (Figure 11C), the MOD group showed a significant elevation compared to the CON group (*p* < 0.05), indicating that ethanol exposure strongly induces CYP2E1 expression, which could lead to enhanced oxidative stress. Notably, H&L treatment significantly reduced CYP2E1 levels compared to the MOD group (*p* < 0.05). This suggests that H&L may suppress CYP2E1-mediated ROS generation, thereby alleviating ethanol-induced oxidative damage.

To sum up, H&L demonstrated a dual regulatory role by enhancing ADH/ALDH activities while suppressing excessive CYP2E1 expression. These findings support its potential for both promoting ethanol clearance and mitigating oxidative stress, providing mechanistic insights into its “anti-hangover” and hepatoprotective properties [39,47].

### 3.9. Alleviating Effects of H&L on Inflammatory Response and Oxidative Stress in Mice

**Figure 12:**
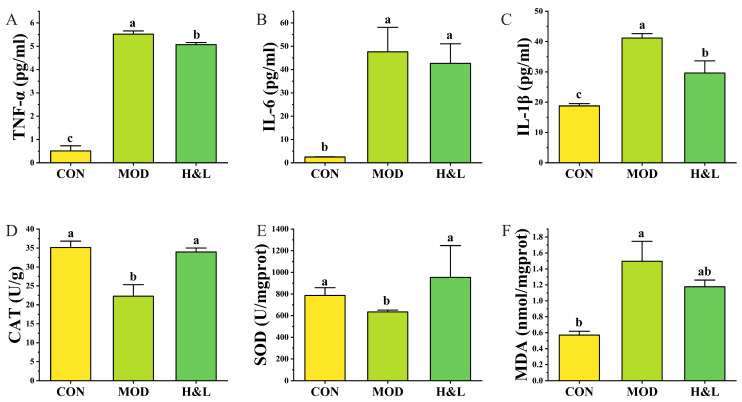
Effects of H&L on serum inflammatory cytokines and hepatic oxidative stress indicators in mice (*n* = 6). (**A**) Serum TNF-α; (**B**) Serum IL-6; (**C**) Serum IL-1β; (**D**) Hepatic CAT activity; (**E**) Hepatic SOD activity; (**F**) Hepatic MDA content. Distinct lowercase letters were used to indicate significant differences between groups.

Previous findings suggested that H&L may influence ethanol-induced inflammation and oxidative stress in vivo. To verify this, we measured the levels of inflammatory cytokines (TNF-α, IL-6, IL-1β) in serum and assessed hepatic antioxidant enzyme activities (CAT, SOD) and the lipid peroxidation product MDA (Figure 12).

In terms of inflammation, serum levels of TNF-α (Figure 12A), IL-6 (Figure 12B), and IL-1β (Figure 12C) were significantly elevated in the MOD group (*p* < 0.05), indicating a strong systemic inflammatory response induced by ethanol. H&L treatment markedly reduced TNF-α and IL-1β levels, with TNF-α significantly lower than in the MOD group (*p* < 0.05), suggesting a superior anti-inflammatory effect. Although the reduction in IL-6 levels was not statistically significant, a downward trend was observed. These results indicate that H&L has a notable alleviating effect on ethanol-induced inflammation.

For oxidative stress, the MOD group showed significantly decreased hepatic CAT (Figure 12D) and SOD (Figure 12E) activities and increased MDA content (Figure 12F), indicating oxidative damage and lipid peroxidation due to ethanol exposure. H&L treatments enhanced CAT and SOD activities and reduced MDA levels. Notably, the H&L group exhibited significantly lower MDA levels compared to the MOD group, indicating a stronger antioxidative effect.

Taken together, H&L effectively attenuates ethanol-induced systemic inflammation, restores antioxidant enzyme activities, and reduces lipid peroxidation, thereby providing dual protection through anti-inflammatory and antioxidant mechanisms [48,49]. These effects are likely associated with the synergistic regulation of the NF-κB pathway and redox balance by the flavonoids, polyphenols, and vitamin C abundantly present in honey and lemon. However, there are still some limitations in our research. For instance, in the results of animal experiments, some indicators, such as liver ethanol content, IL-6, ALDH, etc., although they showed a changing trend, there was no significant difference between the groups. This might be the reason why the ALD symptoms in mice were not significantly relieved due to the short duration of our drug treatment. Another limitation of this study is the absence of a lemon-only group in the animal experiment, which limits the interpretation of the individual contribution of lemon to the observed protective effects of H&L. Furthermore, this study was only verified through animal experiments, which is not sufficient to fully explain the mechanism by which H&L improves ALD. Further verification experiments are needed to verify the reliability of our conclusions.

## 4. Conclusions

This study comprehensively assessed the hepatoprotective effects and underlying mechanisms of H&L against ALD through an integrated approach combining network pharmacology, molecular docking, and animal experiments. A total of 335 potential targets were identified, primarily associated with inflammatory responses, oxidative stress, and ethanol metabolism. In vivo validation suggested that H&L mitigated hepatic injury and was associated with favourable changes in several indicators related to ethanol metabolism, oxidative stress, and inflammation. However, because some variables, including ALT/AST, ALDH, IL-6, and hepatic ethanol content, did not show statistically significant differences, these findings should be interpreted cautiously. Collectively, these findings highlight the potential of H&L as a functional food candidate for ALD prevention and intervention, operating through a synergistic “multi-component–multi-target–multi-pathway” mechanism.

## Figures and Tables

**Table 1:** The docking outcomes including binding energy, number of hydrogen bonds, and key binding residues.

Receptor	Ligand	Binding Energy (kcal/mol)	No. of H-Bonds	Binding Residues
AKT1	Diosmin	−10.8	6	Asp274, Thr82, Tyr18, Asp292, Lys297
AKT1	Eriocitrin	−10.7	8	Asp274, Tyr18, Arg273, Tyr326, Glu17, Asp292, Thr82
AKT1	Hesperidin	−10.8	9	Arg273, Asp274, Asp292, Thr82, Asn54, Tyr326, Glu85, Tyr18
SRC	Diosmin	−11.1	12	Leu273, Met341, Asp404, Lys295, Cys277, Arg388, Asn391, Gln275
SRC	Eriocitrin	−9.6	11	Arg155, Val199, Glu524, Gln526, Pro525, Asp518, Phe520, Glu517, Ser522
SRC	Hesperidin	−11.1	14	Gln275, Asp386, Arg388, Cys277, Lys295, Asn391, Ile336, Met341, Leu273
ADH	Diosmin	−9.8	7	Asn56, Ser54, His51, Glu50, Arg369, Gly202
ADH	Eriocitrin	−9.5	7	Gln271, Arg47, Lys228, Ile269, Va294, His51
ADH	Hesperidin	−9.8	9	Ile269, Gln271, Arg363, Asn225, Lys228, Ser367, Gly202, Arg369
ALDH	Diosmin	−9.9	6	Lys192, Ile166, Glu399, Lys352, Gln196
ALDH	Eriocitrin	−10.2	7	Gln196, Glu195, Gln349, Trp168, Ser246
ALDH	Limonin	−10.1	1	Lys352
CYP2E1	Eriocitrin	−10.0	7	Asp399, Asn400, Ser395, Asp394, Tyr398, Ile361
CYP2E1	Hesperidin	−9.7	9	Asp399, Ser395, His81, Thr58, Asp394, Asn400, Tyr398
CYP2E1	Limonin	−9.6	1	Thr58

## Data Availability

The original contributions presented in this study are included in the article/Supplementary Material. Further inquiries can be directed to the corresponding authors.

## Supplementary Materials

The following supporting information can be downloaded at https://www.mdpi.com/article/10.3390/foods15081384/s1: Table S1. Sugar composition of H&L (g/100 g). Table S2. Physicochemical properties of H&L. Table S3. The main active ingredients of H&L. Table S4. Summary of compound targets. Table S5. 335 common elements in “Disease” and “Drug”. Table S6. The top 15 genes screened by five algorithms based on the cytoHubba plugin. Table S7. The intersection results of the core genes of the five algorithms. Table S8. Receptor protein information for molecular docking. Table S9. Liver pathological scoring criteria. Table S10. 919BPs, 107CCs, and 288MFs from GO enrichment analysis. Table S11. 178 signaling pathways from KEGG enrichment analysis. Table S12. Molecular docking binding energy (kcal/mol).
